# The Relationship Between Mental Health, Disease Severity, and Genetic Risk for Depression in Early Rheumatoid Arthritis

**DOI:** 10.1097/PSY.0000000000000462

**Published:** 2017-06-29

**Authors:** Jack Euesden, Faith Matcham, Matthew Hotopf, Sophia Steer, Andrew P. Cope, Cathryn M. Lewis, Ian C. Scott

**Affiliations:** From the SGDP Centre (Euesden, Lewis), and Department of Psychological Medicine (Matcham, Hotopf), Institute of Psychiatry, Psychology & Neuroscience, King's College London; Department of Rheumatology (Steer), Weston Education Centre, King's College Hospital; Academic Department of Rheumatology (Cope, Scott), Centre for Molecular and Cellular Biology of Inflammation, and Department of Medical and Molecular Genetics (Lewis, Scott), King's College London, London; MRC Integrative Epidemiology Unit (Euesden), School of Social and Community Medicine, University of Bristol, Bristol; and Research Institute for Primary Care & Health Sciences (Scott), Primary Care Sciences, Keele University, Staffordshire, United Kingdom.

**Keywords:** disability, disease activity, genetics, mental health, pain, rheumatoid arthritis, **CARDERA** = Combination Anti-Rheumatic Drugs in Early RA, **CBT** = cognitive-behavioral therapy, **DAS28** = disease activity score on a 28-joint count, **ESR** = erythrocyte sedimentation rate, **GWAS** = genome-wide association study, **HAQ** = health assessment questionnaire, **HRQoL** = health-related quality of life, **HWE** = Hardy-Weinberg equilibrium, **LOCF** = last observation carried forward, **MAF** = minor allele frequency, **MCS** = mental component summary score, **MDD** = major depressive disorder, **MH** = mental health, **PGA** = patient global assessment, ***p***_**T**_ = *p* value threshold, **RA** = rheumatoid arthritis, **RCT** = randomized controlled trial, **REC** = Research Ethics Committee, **RF** = rheumatoid factor, **SE** = standard error, **SF-36** = short form-36, **SJC** = swollen joint count, **SNP** = single nucleotide polymorphism, **TJC** = tender joint count, **VAS** = visual analog scale, **wGRS** = weighted genetic risk score

## Abstract

**Objective:**

Reduced mental health (MH) is prevalent in rheumatoid arthritis (RA). Although longitudinal studies are limited, there is evidence that depression is associated with worse disease outcomes. We evaluated reciprocal relationships between MH, RA severity, and genetic risks for depression for 2 years in a well-characterized cohort of RA patients.

**Methods:**

We evaluated 520 early RA patients previously enrolled to two clinical trials. MH was measured using the short form-36 MH domain and mental component summary scores (MCS). MCS/MH associations over 2 years with disease activity (disease activity score on a 28-joint count), disability (health assessment questionnaire), pain visual analog scale scores, and a weighted genetic risk score for depression were tested using linear mixed-effects and regression models.

**Results:**

Poorer MH was associated with worse RA outcomes. Lower MCS scores (indicating worse MH) were seen in patients with a greater genetic risk for depression (weighted genetic risk score: coefficient = −1.21, *p* = .013). Lower baseline MCS was associated with lower 2-year improvements in disease activity score on a 28-joint count (coefficient = −0.02, *p* < .001), pain (coefficient = −0.33, *p* < .001), and health assessment questionnaire (coefficient = −0.01, *p* = .006). Baseline MCS was associated with changes in the swollen joint count (coefficient = −0.09, *p* < .001) and patient global assessment (coefficient = −0.28, *p* < .001) but not the tender joint count (*p* = .983) and erythrocyte sedimentation rate (*p* = .973). Only baseline pain visual analog scale (coefficient = −0.07, *p* = .002) was associated with 2-year changes in MCS.

**Conclusions:**

Reduced baseline MH was associated with lower improvements in disease activity, disability, and pain for 2 years, supporting current national guidelines recommending screening for depression in RA. Pain had a bidirectional relationship with MH. Depression genetic risk had a significant association with MH.

## Abstract 2

Supplemental digital content is available in the text.

## INTRODUCTION

Reduced mental health (MH) is prevalent in rheumatoid arthritis (RA), with major depression present in 16.8% of patients (1). The cause of this excess burden of MH impairment is uncertain. Comorbid depression also seems to have a detrimental impact on the disease course of RA, being associated with increased healthcare use and costs (2) and representing an independent risk factor for nonsuicide-related mortality (3). Determining the cause and effect of depression in RA is, therefore, a key research goal.

Research in this area has mainly involved cross-sectional studies in patients with long-standing RA. These identified associations between depression and pain (4), disability (5), and arthritis disease activity (6). Their cross-sectional nature, however, made it impossible to infer causality. Although longitudinal studies are limited, there is some evidence for a bidirectional effect with pain in patients with musculoskeletal disorders, whereby depression influences pain and vice versa (7,8). There is also some evidence that depression predicts the subsequent disease activity of RA, with an analysis of established RA patients finding a slower rate of decline in disease activity over time in patients with a history of depression (9).

Depression also has a substantial genetic component (10), with several variants associated with depression identified in a genome-wide association study (GWAS) (11). This is consistent with previous twin studies of the heritability of major depressive disorder (MDD), which found that the disorder has a heritability of 48% to 75%, depending on assumptions made on the prevalence of MDD in the general population (12). The role of these in determining MH in RA has not previously been evaluated.

The aim of our study was to evaluate the relationship between MH and disease activity, disability, pain, and genetic risk for depression for 2 years in a well-characterized clinical trial cohort of patients with early RA. The direction of any associations was tested by examining the impact of baseline MH on changes in disease activity, disability and pain, and vice versa.

## METHODS

### Participants

We studied patients in the Combination Anti-Rheumatic Drugs in Early RA (CARDERA) genetics cohort. It has been described in detail previously (13). In brief, it comprises European ancestry RA patients enrolled to two multicenter randomized controlled trials (RCTs): CARDERA-1 and CARDERA-2 (14,15). Both recruited patients with early RA (<2-year duration) and active disease defined as three of three or more swollen joints, six or more tender joints, 45-minute or more morning stiffness, or erythrocyte sedimentation rate (ESR) of 28 mm/h or greater. CARDERA-1 recruited patients between 2000 and 2002; CARDERA-2 recruited patients between 2003 and 2010. CARDERA-1 randomized patients to receive either (1) methotrexate; (2) methotrexate and ciclosporin; (3) methotrexate and prednisolone; or (4) methotrexate, ciclosporin, and prednisolone. CARDERA-2 randomized patients to receive either (1) methotrexate or (2) methotrexate and anakinra. Because the original aim of the CARDERA studies was to investigate the performance of combination therapy with reference to monotherapy, a placebo group was not assigned. Rheumatoid factor (RF), a biomarker providing clinical information on the antibody composition of patient serum, was assayed as described previously (16). Follow-up was 2 years. The current analysis is restricted to the 520 patients with baseline MH data available.

### Disease Outcomes

The following disease outcomes were captured. First, disease activity (how active a patient's arthritis is) was recorded using the disease activity score on a 28-joint count (DAS28). This composite score combines information on the number of swollen and tender joints (assessed by a clinician from 28 joint counts), the patient global assessment (PGA) of disease activity (which involves a patient rating his or her overall disease activity on a 100-mm visual analog scale [VAS]), and the ESR in a mathematical formula to give an assessment of RA activity ranging from 0 to 10. Lower scores indicate less active disease, with scores of higher than 5.1, lower than 3.2, and lower than 2.6 indicating high disease activity, low disease activity, and remission, respectively. Second, disability was recorded using the health assessment questionnaire (HAQ), a patient-completed questionnaire giving a score of function ranging from 0 to 3. HAQ scores of lower than 1, 1 to 2, and higher than 2 indicate mild, moderate, and severe disability, respectively. Third, pain was recorded using a 100-mm patient completed pain VAS, a method for quantifying the severity of self-reported pain (17). Fourth, health-related quality of life (HRQoL) was recorded using the short form-36 (SF-36), which is described in detail hereinafter. In CARDERA-1, the aforementioned outcomes were captured every 6 months. In CARDERA-2, they were captured at 0, 6, 12, and 24 months.

### Mental Health

The SF-36 is a generic measure of health status, capturing HRQoL across eight domains (4 physical and 4 mental) (18). These domains are scored from 0 to 100, with higher scores indicating better HRQoL. They can be normalized, *z*-transformed, and combined into mental component summary (MCS) and physical component summary scores providing summary measures of overall mental and physical health, relative to a population mean score of 50 (SD = 10) (19).

We used the MH domain score and MCS as measures of MH in our analysis. Both have been used to screen for depression, with an MCS cutoff of 42 having a sensitivity and specificity of 74% and 81%, respectively, for detecting depressive disorder (20). They also associate with depression severity, both cross-sectionally and over time (21).

### Genotyping

CARDERA patients were genotyped on the Illumina ImmunoChip array (described in detail previously (13)). Single nucleotide polymorphism (SNP) markers were removed that had more than 5% missingness, were duplicates, were not in Hardy-Weinberg equilibrium (HWE, *p* < .00001), and had a minor allele frequency (MAF) of less than 0.01. From 196,524 pre–quality control markers, 138,873 were available in the final data set. Imputation was subsequently performed using IMPUTE2 (22) and the 1000 Genomes Phase 1 integrated variant version 3 (March 2012) reference panel (variants filtered with a European MAF <0.01). Postimputation SNPs were removed with low INFO scores (<0.7), MAF (<0.05), HWE (*p* < .000001), and genotyping rate (<0.1), resulting in 429,193 available markers.

### Genetic Risk for Depression

The Psychiatric Genomics Consortium MDD GWAS mega-analysis failed to find a locus of genome-wide significance, likely reflecting limited power caused by the genetic architecture of MDD (small effect sizes of individual genetic variants) and the high prevalence of MDD, which increases the difficulty in recruiting large samples of screened, low-risk controls (23). We therefore tested a weighted genetic risk score (wGRS), combining loci of nominal association with MDD for an association with MH in CARDERA. This approach is commonly used in studies of polygenic disorders, whose genetic architecture comprises thousands of very small effect common alleles (24,25). We used a *p* value threshold (*p*_T_) for SNPs to include in the wGRS of 0.05 (representing nominal association with MDD). A continuous wGRS based on MDD GWAS results has been shown to predict depression in independent cohorts, with a *p*_T_ value of .05 demonstrated to generate a wGRS that most strongly predicts MDD risk (26). After linkage disequilibrium pruning, 3010 SNPs were included in the wGRS. The wGRS was generated for each individual in CARDERA by calculating the number of nominally associated risk alleles they carried, weighted by the log odds ratio from the MDD mega-analysis, summed across SNPs.

### Statistical Analysis

#### Associations with MH

Two different modeling approaches were used to evaluate the relationship between MH, RA severity measures, and genetic risk for depression. The first approach established whether MH was associated with either RA severity measures or the wGRS for depressive disorder over time. This used a linear mixed-effects model, which incorporated either MCS or MH measured at each time point as the response variable regressed on the corresponding predictors (wGRS, DAS28 and its components, HAQ, or pain VAS) from each time point. Effect size estimates (coefficients) for predictor variables provided information on the average differences in the MCS or MH score for the 2-year period relative to the average predictor variable score. We have included a level 1 random effect of individual, fitting random intercepts for each individual. These models have a level 2 random effect for time point, modeling deviation from the overall effect of time point on outcome within each individual as random slopes. We specify this correlation structure using the lme4 package in R (27). The following variables were tested for their associations with MCS: age, sex, disease duration, and RF status. Of these, only sex improved the model fit—as determined using a stepwise selection approach, with the optimal model determined using the Akaike information criterion—and thus, only this variable was included as a covariate (Supplementary Tables A.1 and A.2, Supplemental Digital Content 1, http://links.lww.com/PSYMED/A380). The wGRS was standardized to a *z* score to provide interpretable β values. Examination of residuals from a model containing time, sex, and treatment only confirmed a good model fit (Supplementary Figure A.1, Supplemental Digital Content 1, http://links.lww.com/PSYMED/A380), and variance inflation factors calculated for each predictor-ensured multicollinearity between RA outcomes and DAS28 components were not an issue (Supplementary Table A.3, Supplemental Digital Content 1, http://links.lww.com/PSYMED/A380).

The second approach evaluated the direction of associations between MH and RA severity by testing whether MH at study baseline was associated with 2-year changes in RA outcomes over time or vice versa. This used linear regression models to look at the association between (*a*) baseline MCS or MH and 2-year changes in RA severity measures and (*b*) baseline RA severity measures and 2-year changes in MH scores. These models included the baseline response variable score, treatment, and sex as covariates. Examination of model residuals confirmed good model fits (Supplementary Figure A.1, Supplemental Digital Content 1, http://links.lww.com/PSYMED/A380).

#### Missing Data Imputation

In the original CARDERA-1 trial, missing data at each time point had previously been imputed using last observation carried forward (LOCF) analysis for study end points (DAS28, HAQ, and SF-36). LOCF is a commonly used procedure to address missing data in clinical trials with repeated measures over multiple time points. For each individual, missing values at a time point are replaced by the last observed value of that variable (14,28). In the original CARDERA-2 trial, missing data were not imputed (15). For consistency in the current analysis, we imputed missing, previously nonimputed CARDERA-1 data (swollen joint count [SJC], tender joint count [TJC], ESR, PGA, pain VAS) and missing CARDERA-2 data using LOCF. The largest amount of missing data was seen for pain VAS (11% of observations missing across all time points). We repeated our analysis with nonimputed data only; this excluded a significant impact of the LOCF assumption (Supplementary Table A.4, Supplemental Digital Content 1, http://links.lww.com/PSYMED/A380).

#### Statistical Software

Analyses were performed in the statistical environment R (R Foundation for Statistical Computing, Vienna, Austria), PRSice (Version 1.2) (29), IMPUTE2 (22), and PLINK (Version 1.9) (30).

### Ethics, Consent, and Permissions

CARDERA-1 (South Thames Multicentre Research Ethics Committee (REC) reference: MREC (1) 99/04) and CARDERA-2 (South East REC reference: MREC 02/1/089) were ethically approved. Approval was obtained to genotype archived DNA (National Research Ethics Service Committee East of England-Essex REC reference: 11/EE/0544). All patients provided consent.

## RESULTS

### Patient Baseline Characteristics

Most patients were female (69%, Table 1) and possessed RF in their serum (67%). Baseline disease activity was high (mean DAS28 = 5.88), moderate disability (mean HAQ = 1.56), and short disease duration (mean duration = 3.3 months). Baseline MH was reduced relative to the general population (mean MCS score = 40.6, which is 9.4 U lower than the general population mean).

**TABLE 1:**
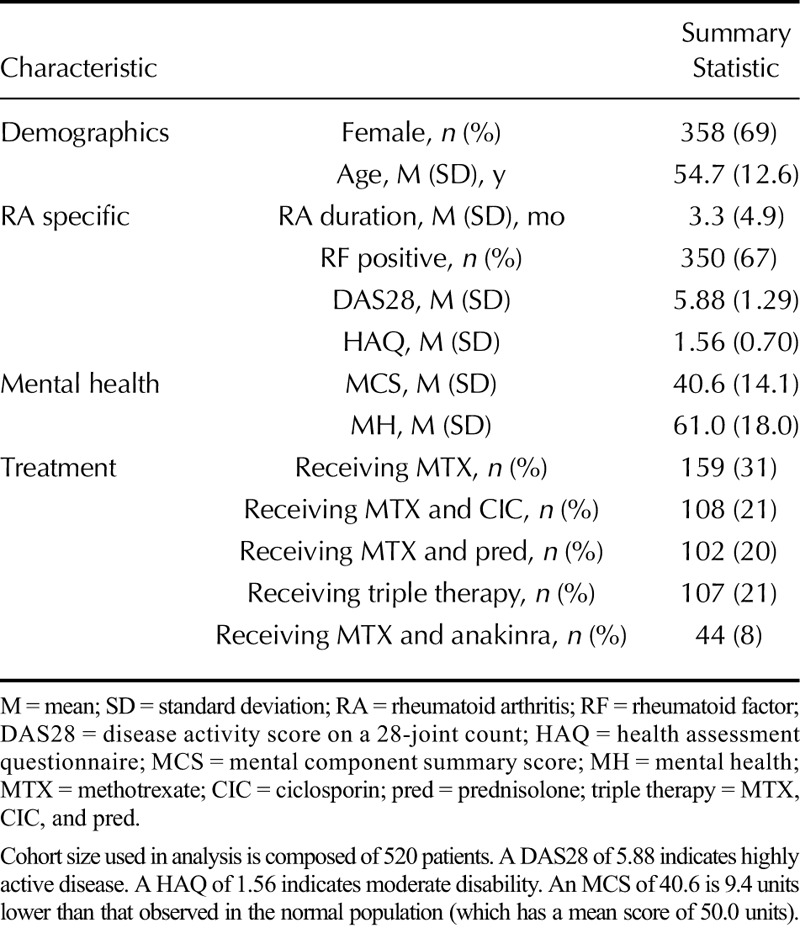
CARDERA Genetics Cohort Baseline Characteristics

### Disease Severity Associations With MH

In a sex- and treatment-adjusted linear mixed-effects model, DAS28 (*p* < .001), HAQ (*p* < .001), and pain VAS (*p* < .001) were significantly associated with MCS (Table 2). On average, for 2 years, MCS scores were 2.22, 6.07, and 0.14 units lower per unit increase in DAS28, HAQ, and pain VAS scores, respectively. This indicates that the higher a patient's disease activity, disability, and pain levels, the worse their MH. In multivariate models, the following three disease severity measures retained a highly significant association with MCS (Table 2): HAQ (coefficient = −3.88, *p* < .001), DAS28 (coefficient = −0.91, *p* < .001), and pain VAS (coefficient = −0.05, *p* < .001). Similar associations were seen with the MH domain.

**TABLE 2:**
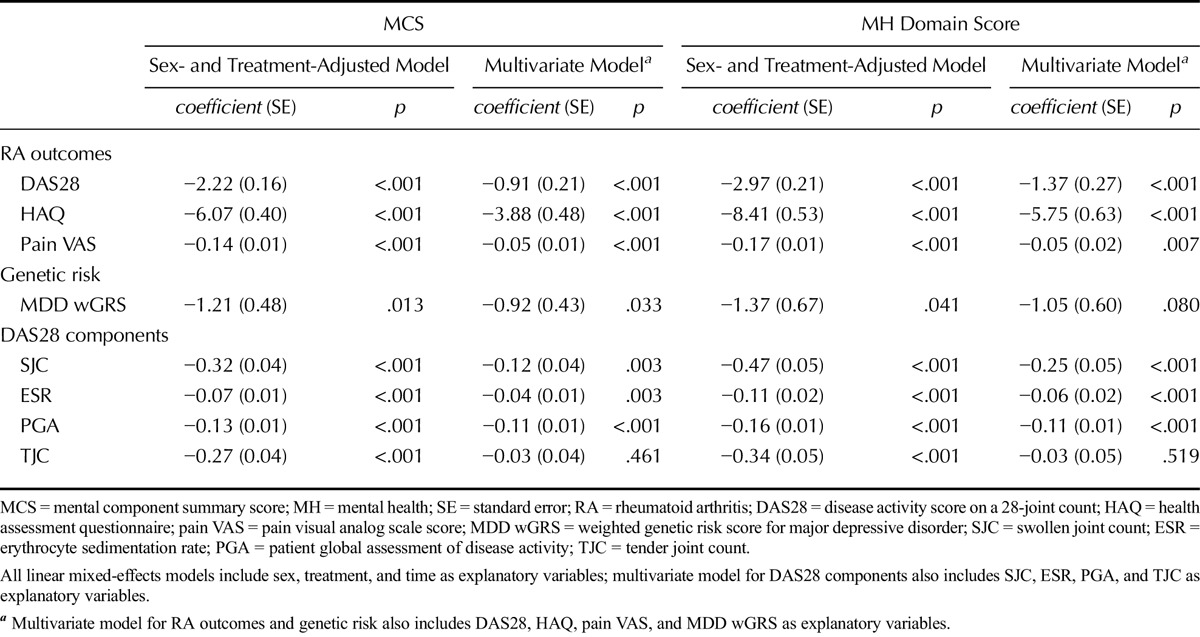
Longitudinal Associations Between MH, RA Outcomes, and Depression Genetic Risk Score

### Major Depressive Disorder Genetic Risk Score Associations With MH

A significant association was seen between the wGRS for depression and MCS (*p* = .013) and MH (*p* = .041) (Table 2). The association with MCS (*p* = .033) but not MH (*p* = .080) was retained in multivariate models including DAS28, HAQ, and pain VAS as covariates. Higher wGRS scores, which indicate a greater genetic risk for depression, were associated with worse MH (lower MCS and MH scores) (MCS: coefficient = −1.21; MH: coefficient = −1.37). Repeating the analysis with a linear mixed-effects model that incorporated a wGRS-time interaction term provided some evidence that genetic risk for depression also predicted the rate at which MH improved across time points, with a significant association seen between the wGRS-time term and MH (*p* = .039, coefficient = −0.83) but not MCS (*p* = .330, coefficient = −0.30).

### Disease Activity Score on a 28-Joint Count Component Associations With MH

In a sex- and treatment-adjusted linear mixed-effects model, all four DAS28 components—SJC, TJC, ESR, and PGA—were associated with MCS and MH scores when tested individually (Table 2). Higher scores in each DAS28 component were associated with lower MCS and MH scores; this indicates that more active disease is linked with poorer MH. On average, for 2 years, MCS scores were 0.32, 0.07, 0.13, and 0.27 units lower per unit increase in SJC, ESR, PGA, and TJC scores, respectively. In multivariate models including all four DAS28 components, the TJC failed to retain a significant association with MCS (*p* = .461) and MH (*p* = .519).

### Direction of Association Between RA Outcomes and MH

#### Association Between Baseline Disease Severity and Changes in MH

The only baseline RA severity measure that had a significant association with 2-year changes in both MCS and MH scores was pain VAS (Table 3). Higher baseline pain VAS scores (indicating greater levels of pain) were associated with lesser increases in MCS and MH scores (indicating lower improvements in MH). The increase in MCS was 0.07 units less per 1-mm increase in baseline pain VAS. A significant association between the baseline TJC and 2-year changes in MH domain scores was also seen (*p* = .023), although this variable was not significantly associated with 2-year changes in MCS scores (*p* = .122).

**TABLE 3:**
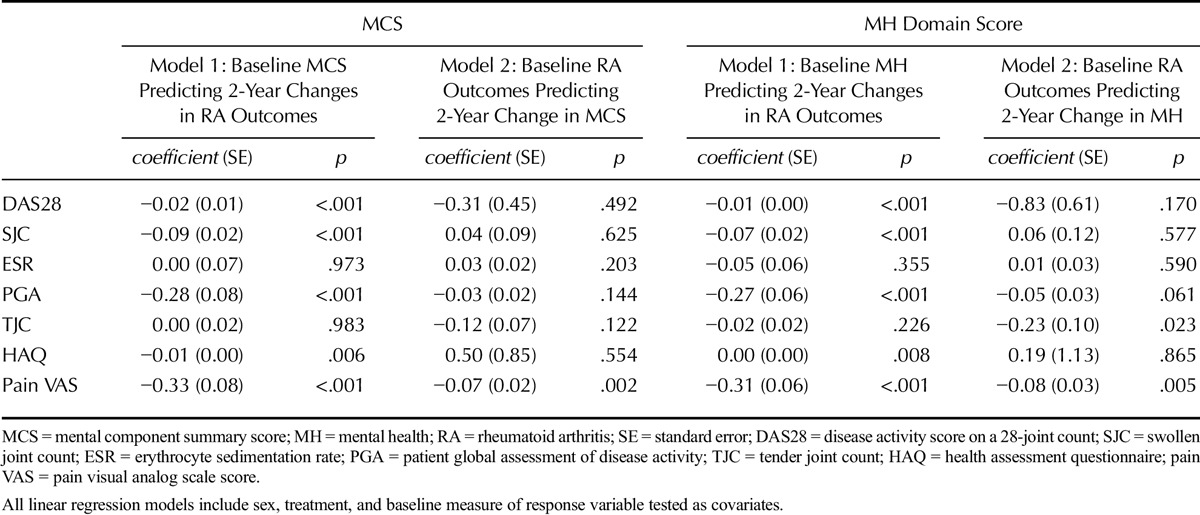
Direction of Associations Between MH and RA Outcomes

#### Association Between Baseline MH and Changes in RA Outcomes

Baseline MCS and MH domain scores had significant inverse associations with 2-year changes in DAS28 (MCS and MH, *p* < .001), pain VAS (MCS and MH, *p* < .001), and HAQ (MCS, *p* = .006; MH, *p* = .008) (Table 3). Lower baseline MCS and MH scores (indicating poorer MH) were associated with lesser improvements in DAS28, pain VAS, and HAQ scores. The effect sizes were, however, modest: per 10 unit increase in baseline MCS, the 2-year reductions in DAS28, HAQ, and pain VAS were 0.20, 0.10, and 3.30 units greater, respectively (Table 3).

**FIGURE 1:**
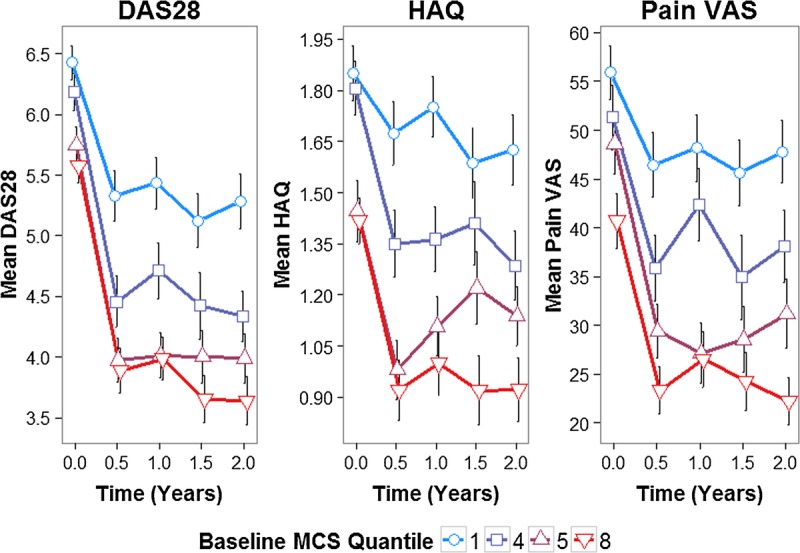
Mean DAS28, HAQ, and pain VAS stratified by baseline MCS octile. MCS divided into octiles (8 quantiles); mean scores with SE bars for octiles 1, 4, 5, and 8 plotted at each time point; to facilitate visual interpretation, octiles 2, 3, 6, and 7 are not plotted, although the same trends are observed (Supplementary Figure A.2, Supplemental Digital Content 1, http://links.lww.com/PSYMED/A380). Color image is available only in online version (www.psychosomaticmedicine.org).

Dividing patients into octiles based on their baseline MCS and plotting the mean disease severity measure in each octile demonstrated the effect of baseline MCS on RA outcomes (Figure 1). Trends toward (*a*) worse disease outcomes at each time point and (*b*) lower improvements in disease outcomes for 2 years across increasing baseline MCS octiles were seen (Figure 1). For 2 years, the mean DAS28, HAQ, and pain VAS scores changed by −1.14, −0.23, and −8.11 U, respectively, in the lowest MCS octile (group 1) and −1.94, −0.49, and −18.49 units, respectively, in the highest MCS octile (group 8).

**FIGURE 2:**
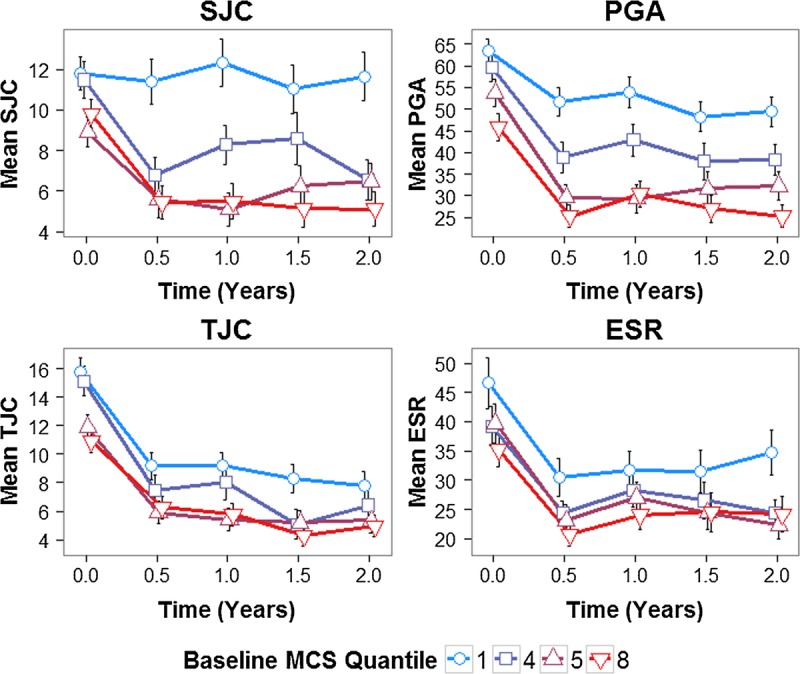
Mean DAS28 components stratified by baseline MCS octile. MCS divided into octiles (8 quantiles); mean scores with SE bars for octiles 1, 4, 5, and 8 plotted at each time point; to facilitate visual interpretation, octiles 2, 3, 6, and 7 are not plotted, although the same trends are observed (Supplementary Figure A.3, Supplemental Digital Content 1, http://links.lww.com/PSYMED/A380). Color image is available only in online version (www.psychosomaticmedicine.org).

Examining individual DAS28 components revealed that baseline MCS and MH scores had significant inverse associations with 2-year changes in the SJC (MCS and MH, *p* < .001) and PGA (MCS and MH, *p* < .001) but not the TJC (MCS, *p* = .983; MH, *p* = .226) and ESR (MCS, *p* = .973; MH, *p* = .355) (Table 3). This differential impact on DAS28 components is shown in Figure 2. For 2 years, the mean SJC, PGA, TJC, and ESR levels changed by −0.17, −13.91, −8.02, and −11.98 units, respectively, in the lowest MCS octile (group 1) and −4.69, −20.46, −5.97, and −11.03, respectively, in the highest baseline MCS octile (group 8).

## DISCUSSION

Our study evaluated the relationship between MH and disease activity, disability, pain, and genetic risk for depression for 2 years in a well-characterized clinical trial cohort of patients with early RA. It has three key findings. The first, and most clinically important, is that low MH was associated with poorer disease outcomes. In a repeated measures analysis, lower MCS and MH scores had significant associations with more active disease, increased disability, and greater pain for 2 years; because MCS and MH scores increased over time, DAS28, HAQ, and pain levels fell. Lower baseline MCS and MH scores (indicating worse MH) were associated with a reduced improvement in disease activity and disability, suggesting that depression predicts the degree to which RA improves over time. The relationship between pain and MH seemed bidirectional, with baseline pain associating with lower improvement in MCS and MH domain scores and vice versa; this is in keeping with existing studies of musculoskeletal disorders (7,8).

The second finding was that swollen, but not TJCs, had a significant association with reduced MH. In a multivariate model incorporating all four DAS28 components, the tender joint component of the DAS28 (TJC) failed to retain a significant association with MCS and MH scores. In established RA patients attending routine clinics, the opposite relationship seems true, with an analysis of the CORRONA registry reporting that a lifetime depression history was associated with slower improvements in the TJC but not the SJC (9). One explanation for the lack of association between MCS/MH scores and the TJC in CARDERA is that the short disease duration of patients means the pain pathway sensitization characterizing fibromyalgic RA—which could be particularly influenced by MH—is yet to occur. An explanation for the association observed between MCS/MH scores and the SJC is that overlapping proinflammatory cytokines, which are present in high levels in early active RA, play important roles in mediating both reduced MH and RA activity. Although this hypothesis is supported by evidence that administering interleukin 1β and tumor necrosis factor α induces depressive behavior in mice (31) and that these cytokines are elevated in the serum of depressed patients (32,33), it fails to explain why baseline MH scores did not predict changes in ESR levels. There is an extensive body of literature investigating the pathophysiology of inflammation-related depression (34), with one proposed mechanism being the activation of the enzyme indoleamine 2,3-dioxygenase by inflammatory cytokines, which catabolizes tryptophan leading to a downstream depletion in serotonin (35)—indeed, inflammation-related depression seems to be dependent on the activation of indoleamine 2,3-dioxygenase (36). In light of this, anti-inflammatory medication has been proposed as a treatment for inflammation-related depression; however, its efficacy is still contested (37); therefore, further research is required in other early active RA cohorts to confirm the generalizability of our results.

Our third finding was that genetic risk for MDD was a significant predictor of MH. We tested a wGRS combining 3010 SNPs of nominal association with MDD in the publicly available Psychiatric Genomics Consortium GWAS for its association with MH in CARDERA. Although a significant association with lower MCS and MH scores was observed, the comparatively large standard error (SE) of the wGRS variable makes any conclusions on its relative importance challenging. The significant interaction term for wGRS-time predicting MH, indicating slower improvements in MH among individuals with high MDD genetic risk, is consistent with previous work indicating that depression genetic risk increases an individual's sensitivity to adverse environmental effects (38,39). Taken together, these findings support the notion that depression is a complex disorder with a modest, albeit important, genetic contribution comprising thousands of alleles of a small effect size.

Our study replicates existing research that depression and pain have a bidirectional relationship. In CARDERA, baseline MCS and MH scores predicted 2-year changes in pain VAS and vice versa. This finding has been documented in psoriatic arthritis; for example, Husted et al. (7) identified a small bidirectional relationship between MCS and pain in 394 patients followed up for a mean of 7.5 years. It has also been reported in patients with persistent back, hip, or knee pain (8), back pain (40,41), and pain from a variety of disorders (42). The complex nature of pain makes it difficult to discern mechanisms by which this pain-depression bidirectional relationship could occur. Possible mechanisms include the following: (1) low mood could affect pain through promoting maladaptive coping strategies, especially catastrophizing (perceiving a situation to be worse than it is) (43); (2) pain could affect MH through reducing daily activities (44) and reducing social activities (45); and (3) imbalances in shared neurotransmitters (serotonin and norepinephrine) in affective and nociceptive pathways could contribute to both mood and pain (44). Further research is required to better characterize the mechanisms underlying this complex relationship.

Supporting our finding that MH predicts disease outcomes across time points, other studies have reported a detrimental impact of reduced MH on patients' responsiveness to anti-inflammatory medication—specifically anti–tumor necrosis factor—as defined by DAS28 change (46,47). This effect is highly relevant to stratified medicine in RA. Although in CARDERA, the impact of baseline MCS on improvements in disease outcomes for 2 years was modest, if considered alongside other poor prognostic markers, such as anti-citrullinated protein antibody status (48), human leukocyte antigen variants (49), smoking, and sex (50), it could provide clinically useful prognostic information, guiding decisions on treatment intensity and facilitating a stratified approach to managing early RA patients.

Our study has several strengths. These include its large size, recruitment from multiple centers spanning two clinical trials, the measurement of multiple disease outcomes in a highly standardized manner, and the short disease duration of RA (mean = 3.3 months), leaving it well placed to examine the effects of MH in very early disease. It also has several weaknesses. As a secondary post hoc analysis of existing RCTs, it did not test a prespecified hypothesis according to a predetermined analysis plan. It evaluated a clinical trial cohort of severe RA patients, limiting its generalizability to patients seen in routine clinical practice. In addition, we only evaluated European ancestry individuals; the relevance of our findings to other ethnic populations is uncertain.

Current National Institute for Health and Care Excellence guidelines for RA management recognize the importance of assessing for comorbid depression, recommending this as part of an annual review process (51). Our findings strongly support this recommendation in early RA. One unresolved issue is the impact of treating depression on the disease course of RA. Although we did not evaluate the impact of MH therapies on RA outcomes, there is some evidence that psychological interventions (such as cognitive behavioral therapy, disclosure therapy, and biofeedback) are useful adjunctive management tools in RA patients. Two systematic literature reviews have evaluated the evidence base for this. Astin et al. (52) reported significant pooled effect sizes for psychological interventions at reducing postinterventional pain, disability, and psychological status across 25 trials. Similarly, Dissanayake and Bertouch (53) found evidence for the efficacy of disclosure therapy and cognitive behavioral therapy with maintenance therapy across four and five studies, respectively. The evidence base is, however, limited with both reviews noting that available trials had methodological limitations. Further research is required to better define the impact of specific psychological interventions at improving disease outcomes in large, well-conducted clinical trials of RA patients.

## CONCLUSIONS

In this cohort of 520 early active RA patients, reduced MH (captured using the SF-36) was associated with worse disease outcomes. Lower MCS and MH scores (indicating poorer MH) were significantly associated with more active disease, increased disability, and greater pain for 2 years. Worse baseline MH was associated with lesser improvements in RA outcome measures, suggesting that depression predicts the rate at which RA improves over time. A bidirectional relationship was observed between MH and pain, replicating existing work in musculoskeletal disorders. Depression genetic risk had a significant, albeit modest, impact on MH. Our findings support the current National Institute for Health and Care Excellence RA management guidelines recommending the annual screening of RA patients for comorbid depression. Further research is needed to establish the impact of specific MH management strategies on improving RA outcomes.

## Supplementary Material

SUPPLEMENTARY MATERIAL

## References

[1] Matcham F Rayner L Steer S Hotopf M The prevalence of depression in rheumatoid arthritis: a systematic review and meta-analysis. Rheumatology (Oxford) 2013;52:2136–48. 24003249 10.1093/rheumatology/ket169 PMC3828510

[2] Joyce AT Smith P Khandker R Melin JM Singh A Hidden cost of rheumatoid arthritis (RA): estimating cost of comorbid cardiovascular disease and depression among patients with RA. J Rheumatol 2009;36:743–52. 19228658 10.3899/jrheum.080670

[3] Ang DC Choi H Kroenke K Wolfe F Comorbid depression is an independent risk factor for mortality in patients with rheumatoid arthritis. J Rheumatol 2005;32:1013–9. 15940760

[4] Kojima M Kojima T Suzuki S Oguchi T Oba M Tsuchiya H Sugiura F Kanayama Y Furukawa TA Tokudome S Ishiguro N Depression, inflammation, and pain in patients with rheumatoid arthritis. Arthritis Rheum 2009;61:1018–24. 19644894 10.1002/art.24647

[5] Peck JR Smith TW Ward JR Milano R Disability and depression in rheumatoid arthritis. A multi-trait, multi-method investigation. Arthritis Rheum 1989;32:1100–6. 2528352 10.1002/anr.1780320908

[6] Cordingley L Prajapati R Plant D Maskell D Morgan C Ali FR Morgan AW Wilson AG Isaacs JD Biologics in Rheumatoid Arthritis Genetics and Genomics Study Syndicate (BRAGGSS) Barton A Impact of psychological factors on subjective disease activity assessments in patients with severe rheumatoid arthritis. Arthritis Care Res (Hoboken) 2014;66:861–8. 24339425 10.1002/acr.22249 PMC4153952

[7] Husted JA Tom BD Farewell VT Gladman DD Longitudinal study of the bidirectional association between pain and depressive symptoms in patients with psoriatic arthritis. Arthritis Care Res (Hoboken) 2012;64:758–65. 22231988 10.1002/acr.21602

[8] Kroenke K Wu J Bair MJ Krebs EE Damush TM Tu W Reciprocal relationship between pain and depression: a 12-month longitudinal analysis in primary care. J Pain 2011;12:964–73. 21680251 10.1016/j.jpain.2011.03.003 PMC3222454

[9] Rathbun AM Harrold LR Reed GW Temporal effect of depressive symptoms on the longitudinal evolution of rheumatoid arthritis disease activity. Arthritis Care Res (Hoboken) 2015;67:765–75. 25384985 10.1002/acr.22515 PMC4886706

[10] Sullivan PF Neale MC Kendler KS Genetic epidemiology of major depression: review and meta-analysis. Am J Psychiatry 2000;157:1552–62. 11007705 10.1176/appi.ajp.157.10.1552

[11] Hyde CL Nagle MW Tian C Chen X Paciga SA Wendland JR Tung JY Hinds DA Perlis RH Winslow AR Identification of 15 genetic loci associated with risk of major depression in individuals of European descent. Nat Genet 2016;48:1031–6. 27479909 10.1038/ng.3623 PMC5706769

[12] McGuffin P Katz R Watkins S Rutherford J A hospital-based twin register of the heritability of DSM-IV unipolar depression. Arch Gen Psychiatry 1996;53:129–36. 8629888 10.1001/archpsyc.1996.01830020047006

[13] Scott IC Rijsdijk F Walker J Quist J Spain SL Tan R Steer S Okada Y Raychaudhuri S Cope AP Lewis CM Do genetic susceptibility variants associate with disease severity in early active rheumatoid arthritis? J Rheumatol 2015;42:1131–40. 25979711 10.3899/jrheum.141211 PMC6714044

[14] Choy EH Smith CM Farewell V Walker D Hassell A Chau L Scott DL CARDERA (Combination Anti-Rheumatic Drugs in Early Rheumatoid Arthritis) Trial Group Factorial randomised controlled trial of glucocorticoids and combination disease modifying drugs in early rheumatoid arthritis. Ann Rheum Dis 2008;67:656–63. 17768173 10.1136/ard.2007.076299

[15] Scott IC Ibrahim F Simpson G Kowalczyk A White-Alao B Hassell A Plant M Richards S Walker D Scott DL A randomised trial evaluating anakinra in early active rheumatoid arthritis. Clin Exp Rheumatol 2016;34:88–93. 26842950

[16] Ma MH Scott IC Dahanayake C Cope AP Scott DL Clinical and serological predictors of remission in rheumatoid arthritis are dependent on treatment regimen. J Rheumatol 2014;41:1298–303. 24931951 10.3899/jrheum.131401

[17] Carlsson AM Assessment of chronic pain. I. Aspects of the reliability and validity of the visual analogue scale. Pain 1983;16:87–101. 6602967 10.1016/0304-3959(83)90088-X

[18] Ware JE Jr. Gandek B Overview of the SF-36 health survey and the international quality of life assessment (IQOLA) project. J Clin Epidemiol 1998;51:903–12. 9817107 10.1016/s0895-4356(98)00081-x

[19] Ware JE Jr. Kosinski M Dewey J How to Score Version 2 of the SF-36 Health Survey (Standard and Acute Forms). Lincoln, RI: Quality Metric, Inc; 2005.

[20] Ware JE Kosinski M Keller SF SF-36 Physical and Mental Health Summary Scales: A User's Manual. Boston, MA: The Health Institute; 1994.

[21] Sturgeon JA Zautra AJ State and trait pain catastrophizing and emotional health in rheumatoid arthritis. Ann Behav Med 2013;45:69–77. 22915012 10.1007/s12160-012-9408-z PMC3547141

[22] Howie BN Donnelly P Marchini J A flexible and accurate genotype imputation method for the next generation of genome-wide association studies. PLoS Genet 2009;5:e1000529. 19543373 10.1371/journal.pgen.1000529 PMC2689936

[23] Major Depressive Disorder Working Group of the Psychiatric GWAS Consortium, Ripke S Wray NR Lewis CM Hamilton SP Weissman MM Breen G Byrne EM Blackwood DH Boomsma DI Cichon S Heath AC Holsboer F Lucae S Madden PA Martin NG McGuffin P Muglia P Noethen MM Penninx BP Pergadia ML Potash JB Rietschel M Lin D Müller-Myhsok B Shi J Steinberg S Grabe HJ Lichtenstein P Magnusson P Perlis RH Preisig M Smoller JW Stefansson K Uher R Kutalik Z Tansey KE Teumer A Viktorin A Barnes MR Bettecken T Binder EB Breuer R Castro VM Churchill SE Coryell WH Craddock N Craig IW Czamara D De Geus EJ Degenhardt F Farmer AE Fava M Frank J Gainer VS Gallagher PJ Gordon SD Goryachev S Gross M Guipponi M Henders AK Herms S Hickie IB Hoefels S Hoogendijk W Hottenga JJ Iosifescu DV Ising M Jones I Jones L Jung-Ying T Knowles JA Kohane IS Kohli MA Korszun A Landen M Lawson WB Lewis G Macintyre D Maier W Mattheisen M McGrath PJ McIntosh A McLean A Middeldorp CM Middleton L Montgomery GM Murphy SN Nauck M Nolen WA Nyholt DR O'Donovan M Oskarsson H Pedersen N Scheftner WA Schulz A Schulze TG Shyn SI Sigurdsson E Slager SL Smit JH Stefansson H Steffens M Thorgeirsson T Tozzi F Treutlein J Uhr M van den Oord EJ Van Grootheest G Völzke H Weilburg JB Willemsen G Zitman FG Neale B Daly M Levinson DF Sullivan PF A mega-analysis of genome-wide association studies for major depressive disorder. Mol Psychiatry 2013;18:497–511. 22472876 10.1038/mp.2012.21 PMC3837431

[24] Cross-Disorder Group of the Psychiatric Genomics Consortium. Identification of risk loci with shared effects on five major psychiatric disorders: a genome-wide analysis. Lancet 2013;381:1371–9. 23453885 10.1016/S0140-6736(12)62129-1 PMC3714010

[25] International Schizophrenia Consortium, Purcell SM Wray NR Stone JL Visscher PM O'Donovan MC Sullivan PF Sklar P Common polygenic variation contributes to risk of schizophrenia and bipolar disorder. Nature 2009;460:748–52. 19571811 10.1038/nature08185 PMC3912837

[26] Peyrot WJ Milaneschi Y Abdellaoui A Sullivan PF Hottenga JJ Boomsma DI Penninx BW Effect of polygenic risk scores on depression in childhood trauma. Br J Psychiatry 2014;205:113–9. 24925986 10.1192/bjp.bp.113.143081 PMC4118052

[27] Bates D Machler M Bolker B Walker S Fitting linear mixed-effects models using lme4. J Statistic Soft 2015;67:48.

[28] Scott IC Ibrahim F Lewis CM Scott DL Strand V Impact of intensive treatment and remission on health-related quality of life in early and established rheumatoid arthritis. RMD Open 2016;2:e000270. 27651924 10.1136/rmdopen-2016-000270 PMC5013499

[29] Euesden J Lewis CM O'Reilly PF PRSice: polygenic risk score software. Bioinformatics 2015;31:1466–8. 25550326 10.1093/bioinformatics/btu848 PMC4410663

[30] Chang CC Chow CC Tellier LC Vattikuti S Purcell SM Lee JJ Second-generation PLINK: rising to the challenge of larger and richer datasets. Gigascience 2015;4:7. 25722852 10.1186/s13742-015-0047-8 PMC4342193

[31] Bluthé RM Pawlowski M Suarez S Parnet P Pittman Q Kelley KW Dantzer R Synergy between tumor necrosis factor alpha and interleukin-1 in the induction of sickness behavior in mice. Psychoneuroendocrinology 1994;19:197–207. 8190839 10.1016/0306-4530(94)90009-4

[32] Owen BM Eccleston D Ferrier IN Young AH Raised levels of plasma interleukin-1beta in major and postviral depression. Acta Psychiatr Scand 2001;103:226–8. 11240580 10.1034/j.1600-0447.2001.00162.x

[33] Tuglu C Kara SH Caliyurt O Vardar E Abay E Increased serum tumor necrosis factor-alpha levels and treatment response in major depressive disorder. Psychopharmacology (Berl) 2003;170:429–33. 12955291 10.1007/s00213-003-1566-z

[34] Miller AH Raison CL The role of inflammation in depression: from evolutionary imperative to modern treatment target. Nat Rev Immunol 2016;16:22–34. 26711676 10.1038/nri.2015.5 PMC5542678

[35] Capuron L Ravaud A Neveu PJ Miller AH Maes M Dantzer R Association between decreased serum tryptophan concentrations and depressive symptoms in cancer patients undergoing cytokine therapy. Mol Psychiatry 2002;7:468–73. 12082564 10.1038/sj.mp.4000995

[36] O'Connor JC André C Wang Y Lawson MA Szegedi SS Lestage J Castanon N Kelley KW Dantzer R Interferon-gamma and tumor necrosis factor-alpha mediate the upregulation of indoleamine 2,3-dioxygenase and the induction of depressive-like behavior in mice in response to bacillus Calmette-Guerin. J Neurosci 2009;29:4200–9. 19339614 10.1523/JNEUROSCI.5032-08.2009 PMC2835569

[37] Miller AH Raison CL Are anti-inflammatory therapies viable treatments for psychiatric disorders?: Where the rubber meets the road. JAMA Psychiatry 2015;72:527–8. 25853989 10.1001/jamapsychiatry.2015.22 PMC5542670

[38] Mullins N Power RA Fisher HL Hanscombe KB Euesden J Iniesta R Levinson DF Weissman MM Potash JB Shi J Uher R Cohen-Woods S Rivera M Jones L Jones I Craddock N Owen MJ Korszun A Craig IW Farmer AE McGuffin P Breen G Lewis CM Polygenic interactions with environmental adversity in the aetiology of major depressive disorder. Psychol Med 2016;46:759–70. 26526099 10.1017/S0033291715002172 PMC4754832

[39] Keers R Coleman JR Lester KJ Roberts S Breen G Thastum M Bögels S Schneider S Heiervang E Meiser-Stedman R Nauta M Creswell C Thirlwall K Rapee RM Hudson JL Lewis C Plomin R Eley TC A genome-wide test of the differential susceptibility hypothesis reveals a genetic predictor of differential response to psychological treatments for child anxiety disorders. Psychother Psychosom 2016;85:146–58. 27043157 10.1159/000444023 PMC5079103

[40] Hurwitz EL Morgenstern H Yu F Cross-sectional and longitudinal associations of low-back pain and related disability with psychological distress among patients enrolled in the UCLA Low-Back Pain Study. J Clin Epidemiol 2003;56:463–71. 12812821 10.1016/s0895-4356(03)00010-6

[41] Meyer T Cooper J Raspe H Disabling low back pain and depressive symptoms in the community-dwelling elderly: a prospective study. Spine 2007;32:2380–6. 17906583 10.1097/BRS.0b013e3181557955

[42] Geerlings SW Twisk JW Beekman AT Deeg DJ van Tilburg W Longitudinal relationship between pain and depression in older adults: sex, age and physical disability. Soc Psychiatry Psychiatr Epidemiol 2002;37:23–30. 11926200 10.1007/s127-002-8210-2

[43] Quartana PJ Campbell CM Edwards RR Pain catastrophizing: a critical review. Expert Rev Neurother 2009;9:745–58. 19402782 10.1586/ERN.09.34 PMC2696024

[44] Bair MJ Robinson RL Katon W Kroenke K Depression and pain comorbidity: a literature review. Arch Intern Med 2003;163:2433–45. 14609780 10.1001/archinte.163.20.2433

[45] Dickens C Creed F The burden of depression in patients with rheumatoid arthritis. Rheumatology (Oxford) 2001;40:1327–30. 11752500 10.1093/rheumatology/40.12.1327

[46] Hider SL Tanveer W Brownfield A Mattey DL Packham JC Depression in RA patients treated with anti-TNF is common and under-recognized in the rheumatology clinic. Rheumatology (Oxford) 2009;48:1152–4. 19608723 10.1093/rheumatology/kep170

[47] Kekow J Moots R Khandker R Melin J Freundlich B Singh A Improvements in patient-reported outcomes, symptoms of depression and anxiety, and their association with clinical remission among patients with moderate-to-severe active early rheumatoid arthritis. Rheumatology (Oxford) 2011;50:401–9. 21059675 10.1093/rheumatology/keq327

[48] Seegobin SD Ma MH Dahanayake C Cope AP Scott DL Lewis CM Scott IC ACPA-positive and ACPA-negative rheumatoid arthritis differ in their requirements for combination DMARDs and corticosteroids: secondary analysis of a randomized controlled trial. Arthritis Res Ther 2014;16:R13. 24433430 10.1186/ar4439 PMC3979097

[49] Viatte S Plant D Han B Fu B Yarwood A Thomson W Symmons DP Worthington J Young A Hyrich KL Morgan AW Wilson AG Isaacs JD Raychaudhuri S Barton A Association of HLA-DRB1 haplotypes with rheumatoid arthritis severity, mortality, and treatment response. JAMA 2015;313:1645–56. 25919528 10.1001/jama.2015.3435 PMC4928097

[50] Scott IC Lewis CM Cope AP Steer S Rheumatoid arthritis severity: its underlying prognostic factors and how they can be combined to inform treatment decisions. Int J Clin Rheumatol 2013;8:247–63.

[51] National Institute for Health and Care Excellence. Rheumatoid arthritis: the management of rheumatoid arthritis in adults. NICE guidelines [CG79]. NICE Web site. Available at: http://www.nice.org.uk/TA130. Accessed February 11, 2015.

[52] Astin JA Beckner W Soeken K Hochberg MC Berman B Psychological interventions for rheumatoid arthritis: a meta-analysis of randomized controlled trials. Arthritis Rheum 2002;47:291–302. 12115160 10.1002/art.10416

[53] Dissanayake RK Bertouch JV Psychosocial interventions as adjunct therapy for patients with rheumatoid arthritis: a systematic review. Int J Rheum Dis 2010;13:324–34. 21199467 10.1111/j.1756-185X.2010.01563.x

