# Supplementary material for: The Relationship Between Mental Health, Disease Severity, and Genetic Risk for Depression in Early Rheumatoid Arthritis
Source: Psychosom Med. 2017 Jun 29;79(6):638–45. doi: 10.1097/PSY.0000000000000462 (PMC5638421; doi:10.1097/PSY.0000000000000462)
Supplement: SUPPLEMENTARY MATERIAL [file psm-79-638-s001.docx]

**THE RELATIONSHIP BETWEEN MENTAL HEALTH, DISEASE SEVERITY AND GENETIC RISK FOR DEPRESSION IN EARLY RHEUMATOID ARTHRITIS**

**SUPPLEMENTARY MATERIAL APPENDICES**

Authors: Jack Euesden, Faith Matcham, Matthew Hotopf, Sophia Steer, Andrew P Cope, Cathryn M Lewis, Ian C Scott

**INDEX**

- Page 3- Table A.1. Selection of Modelling Covariates in Linear Mixed-Effects Model
- Page 4- Table A.2. Associations between Modelling Covariates and MCS in Linear Mixed-Effects Model
- Page 5- Table A.3. Variance Inflation Factors (VIF) for Predictors in Multivariate Linear Mixed-Effects Models
- Page 6- Table A.4. Associations With MCS Using Linear Mixed-Effects Model In Imputed Versus Non-Imputed Data
- Page 7- Figure A.1. Residual Plots from Linear Mixed-Effects and Linear Regression Models
- Page 8. Figure A.2. Mean DAS28, HAQ and Pain VAS Stratified by Baseline MCS Octile
- Page 9. Figure A.3. Mean DAS28 Components Stratified by Baseline MCS Octile

**Table A.1. Selection of Modelling Covariates in Linear Mixed-Effects Model**

| **Variable** | **Elimination Number** | ***P*-Value** |
| --- | --- | --- |
| RF-Positive | 1 | 0.780 |
| Disease Duration | 2 | 0.354 |
| Age | 3 | 0.286 |
| Treatment | kept | 0.027 |
| Gender | kept | 0.001 |
| Time | kept | <0.001 |

A stepwise AIC was used to select modelling covariates that significantly predicted MCS scores over time within a linear mixed-effects model. After three iterations, only treatment, gender and time were significant predictors of MCS and were included as modelling covariates (treatment and gender as fixed-effects, and a random effect of time).

**Supplementary Table A.2. Associations between Modelling Covariates and MCS in Linear Mixed-Effects Model**

| **Variable** | | **Coefficient** | **SE** | **P-Value** |
| --- | --- | --- | --- | --- |
| **Treatment** | *Methotrexate Monotherapy* | Reference | Reference | Reference |
|  | *Ciclosporin-Methotrexate* | -2.50 | 1.37 | 0.069 |
|  | *Prednisolone-Methotrexate* | -0.32 | 1.40 | 0.818 |
|  | *Methotrexate-Prednisolone-Ciclosporin* | 2.27 | 1.37 | 0.099 |
|  | *Methotrexate-Anakinra* | 1.52 | 1.89 | 0.421 |
| **Gender** |  | 3.35 | 1.04 | 0.001 |
| **Time** |  | 1.71 | 0.31 | <0.001 |

Model includes MCS as response variable and treatment, gender and time as explanatory variables.

**Table A.3. Variance Inflation Factors (VIF) for Predictors in Multivariate Linear Mixed-Effects Models**

**A: VIF for RA severity metrics**

| **Variables** | **VIF** |
| --- | --- |
| *HAQ* | 1.55 |
| *DAS28* | 2.01 |
| *Pain* | 1.76 |
| *wGRS* | 1.02 |

Model includes MCS as response variable and treatment, gender, time, HAQ, DAS28, Pain VAS, and wGRS as explanatory variables.

**B: VIF for DAS components**

| **Variables** | **VIF** |
| --- | --- |
| *ESR* | 1.12 |
| *TJC* | 1.51 |
| *SJC* | 1.41 |
| *PGA* | 1.51 |

Model includes MCS as response variable and treatment, gender, time, ESR, TJC, SJC, and PGA as explanatory variables.

**Table A.4. Associations With MCS Using Linear Mixed-Effects Model In Imputed Versus Non-Imputed Data**

|  | **Non-Imputed** | | **Imputed** |  |
| --- | --- | --- | --- | --- |
| **Variable** | **Coefficient** | ***P*-value** | **Coefficient** | ***P*-value** |
| TJC | -0.28 | <0.001 | -0.27 | <0.001 |
| SJC | -0.35 | <0.001 | -0.32 | <0.001 |
| ESR | -0.07 | <0.001 | -0.07 | <0.001 |
| PGA | -0.14 | <0.001 | -0.13 | <0.001 |
| HAQ | -6.10 | <0.001 | -6.07 | <0.001 |
| Pain | -0.14 | <0.001 | -0.14 | <0.001 |
| DAS28 | -2.25 | <0.001 | -2.22 | <0.001 |

To ensure LOCF imputation had not biased our results we repeated the analysis using non-imputed data. The results are highly similar, confirming the LOCF assumpation was met. A linear mixed-effects models is used in this analysis, including MCS as the response variable and time, treatment, gender and each RA outcome as an explanatory variable in seven separate models (one per RA outcome).

**Figure A.1. Residual Plots from Linear Mixed-Effects and Linear Regression Models**

**
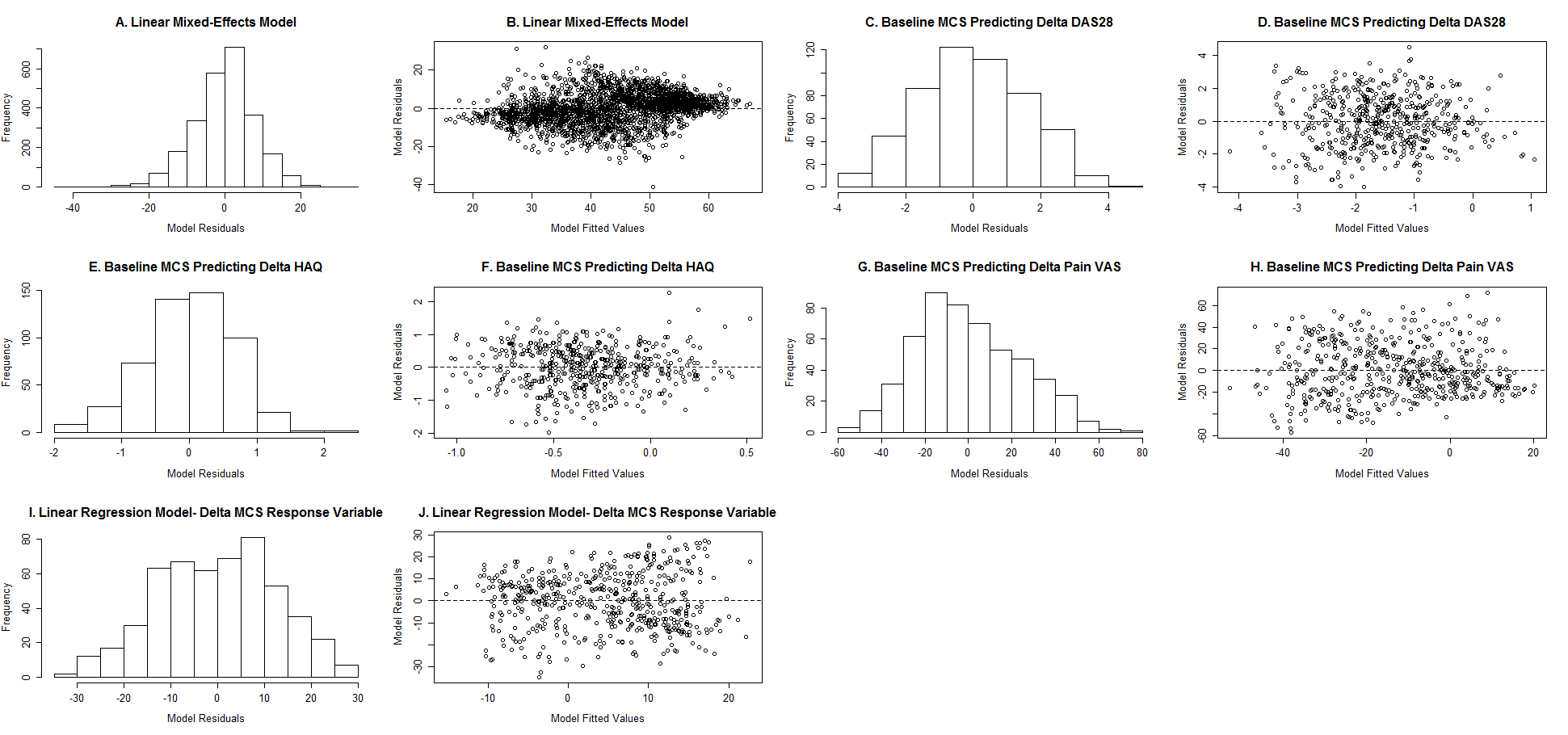
**

A, C, E, G and I are histograms of residuals from each model; B, D, F, H and J are plots of the fitted values against the residuals from each model; A and B = linear mixed-effects model including MCS as the response variable and gender, treatment and time as explanatory variables; C and D = linear regression model including 2-year change in DAS28 as the response variable and baseline MCS, baseline DAS28, gender and treatment as explanatory variables; E and F = linear regression model including 2-year change in HAQ as the response variable and baseline MCS, baseline HAQ, gender and treatment as explanatory variables; G and H = linear regression model including 2-year change in pain VAS as the response variable and baseline MCS, baseline pain VAS, gender and treatment as explanatory variables; I and J = linear regression model including 2-year change in MCS as response variable and baseline MCS, gender and treatment as explanatory variables.

**Figure A.2. Mean DAS28, HAQ and Pain VAS Stratified by Baseline MCS Octile**


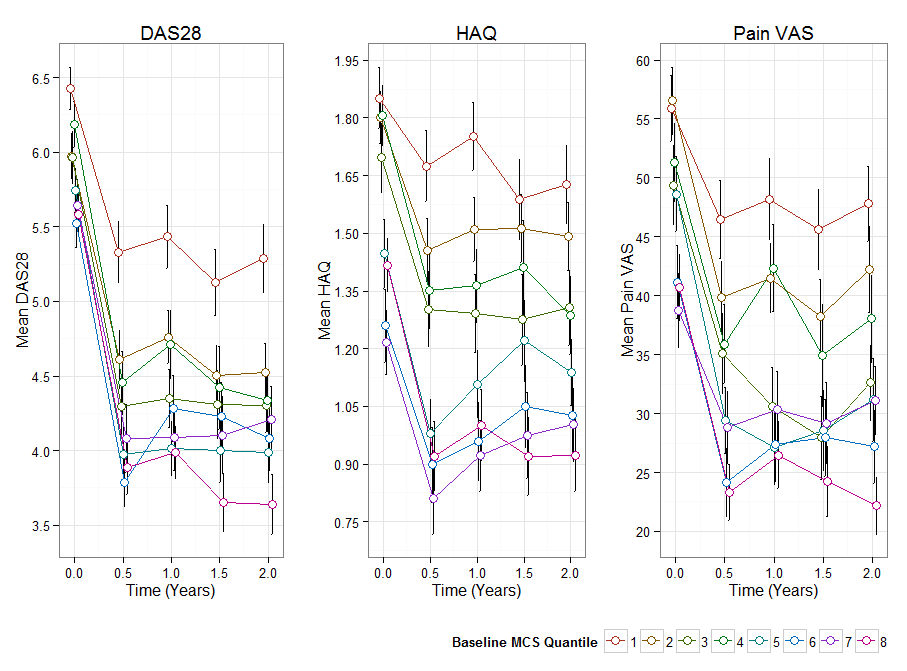


MCS divided into octiles (8 quantiles); mean scores with standard error bars for octiles are plotted at each time point.

**Figure A.3. Mean DAS28 Components Stratified by Baseline MCS Octile**


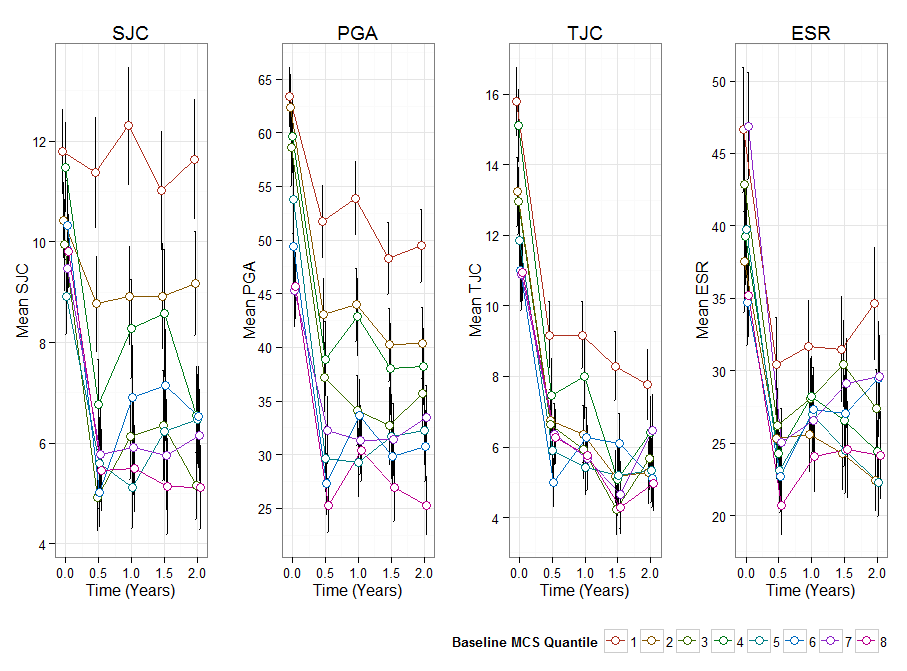


MCS divided into octiles (8 quantiles); mean scores with standard error bars for octiles are plotted at each time point.
